# Supplementary material for: Nuclear receptor corepressor 1 represses cardiac hypertrophy
Source: EMBO Mol Med. 2019 Sep 18;11(11):e9127. doi: 10.15252/emmm.201809127 (PMC6835202; doi:10.15252/emmm.201809127)
Supplement: Supplementary file 1 — Appendix [file EMMM-11-e9127-s001.pdf]

Table of Content

Nuclear Receptor Corepressor 1 Represses Cardiac Hypertrophy
Running Title: NCoR1 represses cardiac hypertrophy

Chao Li† [1, 2, 3], Xue-Nan Sun† [1, 2, 4], Bo-Yan Chen†[1, 2], Meng-Ru Zeng†[1, 2, 4], Lin-Juan Du[1, 2, 4], Ting Liu[1, 2], Hui-Hui Gu[5], Yuan Liu[1, 2, 4], Yu-Lin Li[1, 2], Lu-Jun Zhou[1, 2], Xiao-Jun Zheng[1, 2, 4], Yu-Yao Zhang[1, 2, 4], Wu-Chang Zhang[1, 2], Yan Liu[1, 2], Chaoji Shi[1, 2], Shuai Shao[6], Xue-Rui Shi[7], Yi Yi[7], Xu Liu[7], Jun Wang[5], Johan Auwerx[8], Zhao V. Wang[3], Feng Jia*[6], Ruo-Gu Li*[7], Sheng-Zhong Duan*[1, 2]

Appendix	Page
Appendix Figure S1	2
Appendix Figure S2	3
Appendix Figure S3	4
Appendix Table S1	5
Appendix Table S2	6
Appendix Table S3	7
Appendix Table S4	8
Appendix Table S5	9

Appendix Figure S1

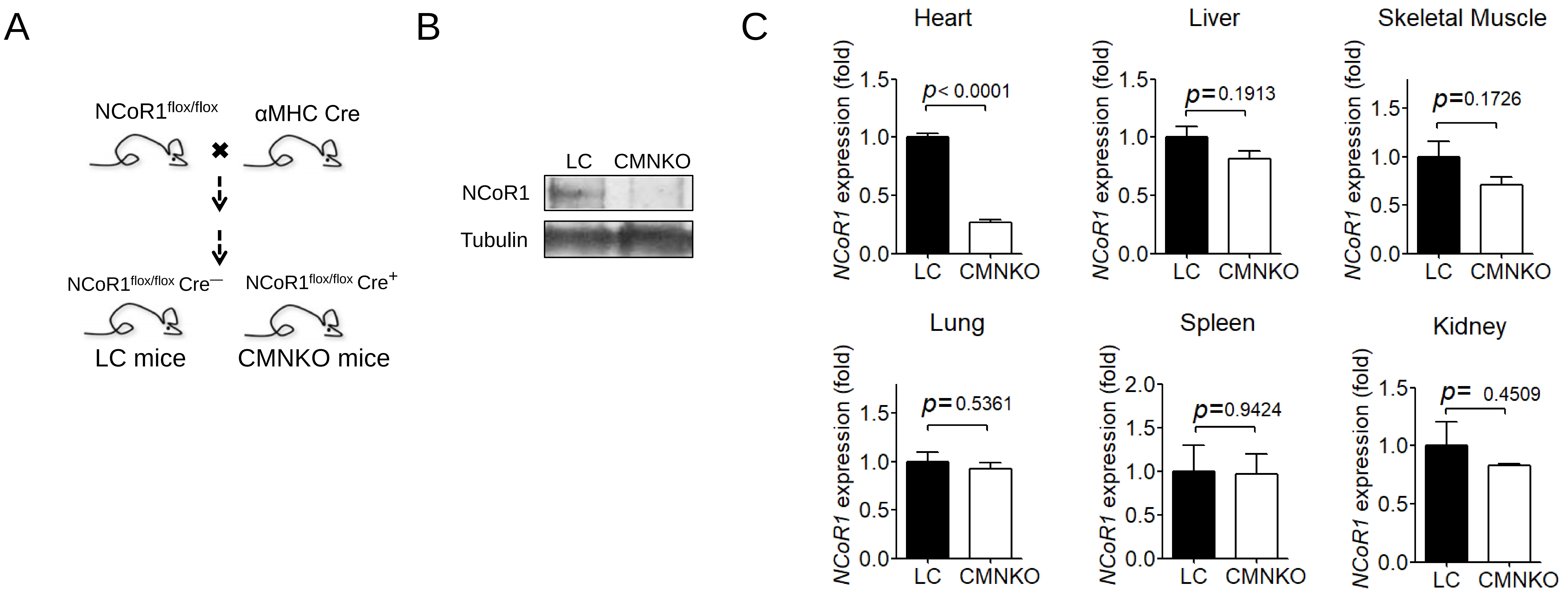

Appendix Figure S1. Generation of cardiomyocyte-specific NCoR1 knockout mice. (A) Schematic illustration of the strategy to generate cardiomyocyte-specific NCoR1 knockout (CMNKO) mice. **(B)** Western blotting analysis of NCoR1 in ventricular samples of littermate control (LC) or CMNKO mice. **(C)** QRT-PCR analysis of NCoR1 in different tissues from LC or CMNKO mice. n=8:7 for Heart, n=3:3 for other tissues. Student's t test was used for statistical analysis.

Appendix Figure S2

A

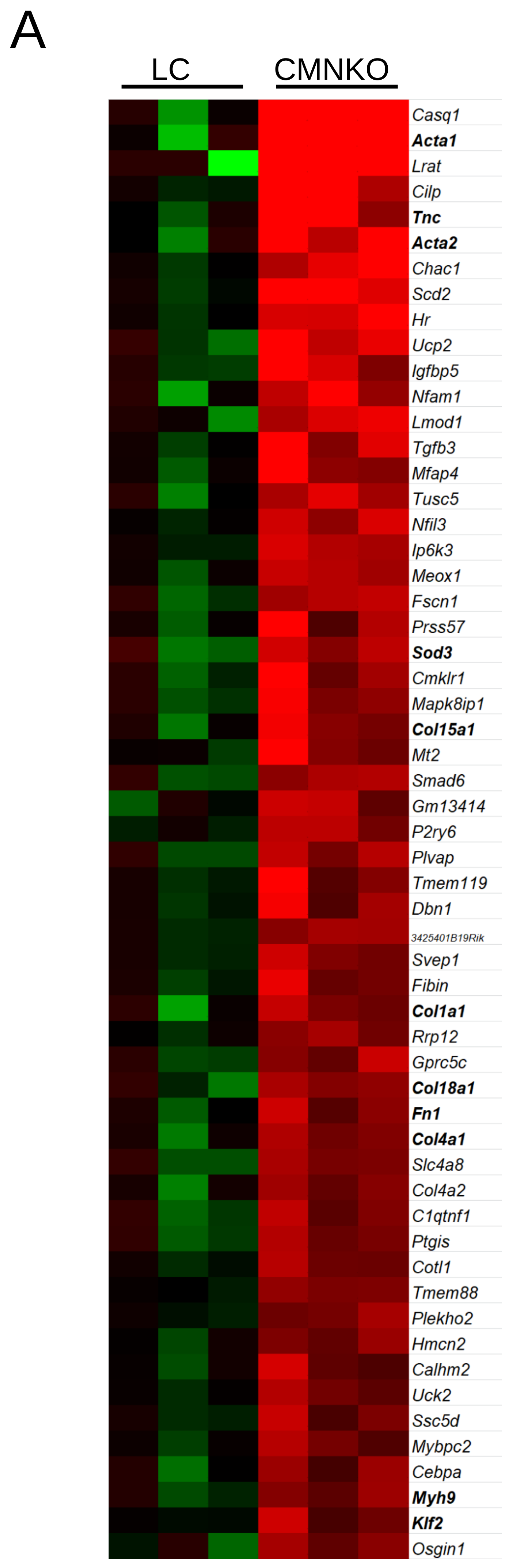

B

AccID	KeggID	Description	Reference
Acta1	mmu:11459	Actin, alpha skeletal muscle	*Biochim Biophys Acta. 2013 Dec;1832(12):2414-24.*
Tnc	mmu:21923	Tnc protein	*Dev Biol. 2009 Apr 15;328(2):392-402.*
Acta2	mmu:11475	Actin, aortic smooth muscle	*Gastroenterology. 2004 Oct;127(4):1174-88.*
Sod3	mmu:20657	Extracellular superoxide dismutase [Cu-Zn]	*Free Radic Res. 2018 Jul;52(7):799-807.*
Col15a1	mmu:12819	Collagen alpha-1(XV) chain	*Sci Rep. 2017; 7: 4476.*
Col1a1	mmu:12842	Collagen alpha-1(I) chain	*Gastroenterology. 2004 Oct;127(4):1174-88.;J Biol Chem. 2006 Apr 7;281(14):9152-62*
Col18a1	mmu:12822	Collagen alpha-1(XVIII) chain	*Sci Rep. 2017; 7: 4476.;J Biol Chem. 2006 Apr 7;281(14):9152-62*
Fn1	mmu:14268	Fibronectin	*J Biol Chem. 2006 Apr 7;281(14):9152-62*
Col4a1	mmu:12826	Collagen alpha-1(IV) chain	*J Biol Chem. 2006 Apr 7;281(14):9152-62*
Myh9	mmu:17886	Myh9 protein	*BMC Bioinformatics. 2009 Sep 17;10 Suppl 9:S5.*
Klf2	mmu:16598	Putative uncharacterized protein	*Blood. 2010 Apr 8;115(14):2971-9.*

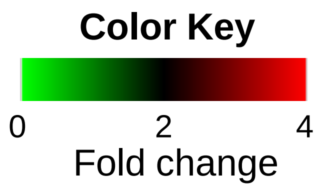

C

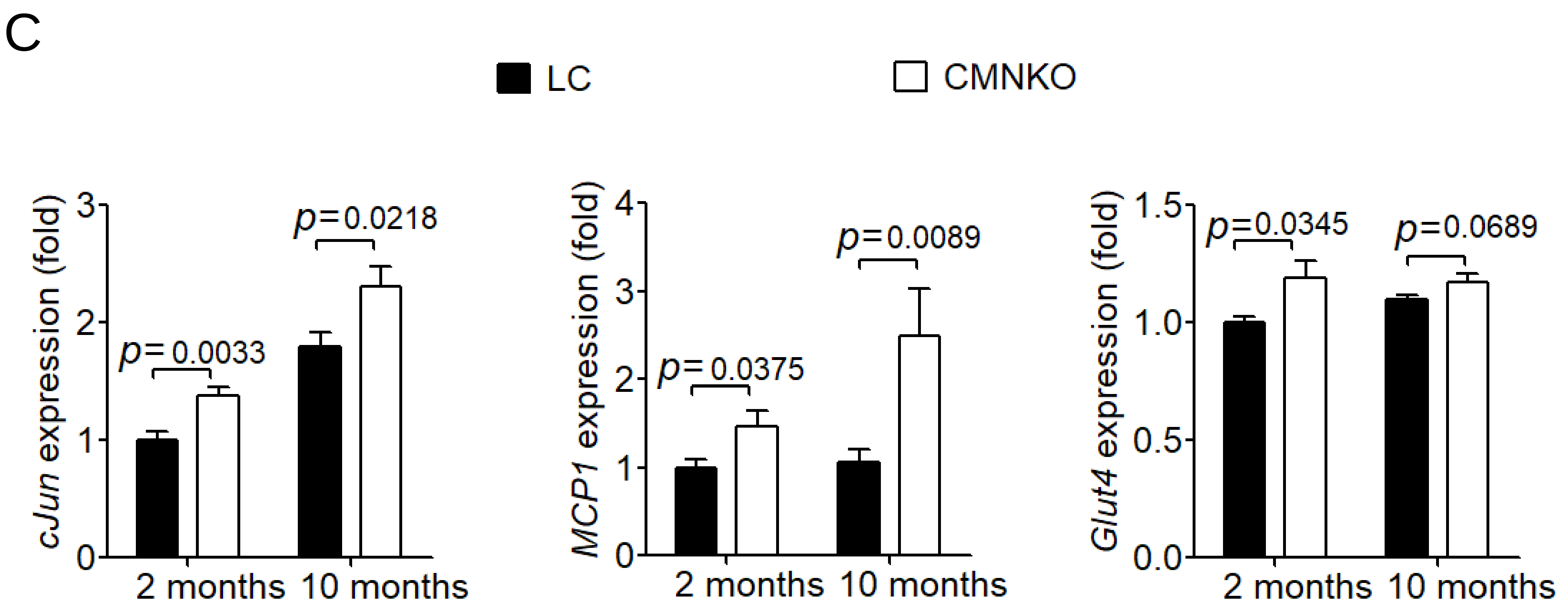

Appendix Figure S2. RNA-sequencing analysis of ventricular samples from LC and CMNKO mice. (A) Heat-map illustration of 57 genes upregulated by cardiomyocyte NCoR1 deficiency. Fold change>2, FDR<0.05, n=3. **(B)** List of MEF2-regulated genes selected from (A). **(C)** qRT-PCR analysis of cJun, monocyte chemoattractant protein 1 (MCP1), and glucose transporter type 4 (Glut4) in left ventricles. n=9:9:11:8. Student's t test was used for statistical analysis.

Apendix Figure S3

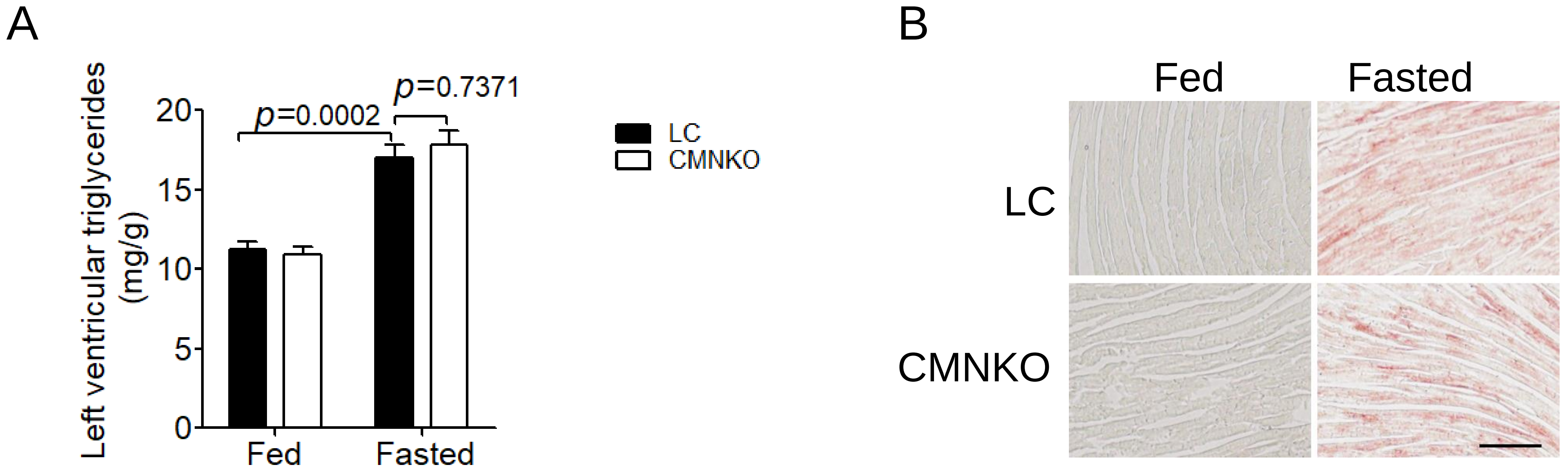

Appendix Figure S3. NCoR1 does not affect the accumulation of triglycerides under fed or fasted condition. (A) Myocardial triglyceride contents of 2-month-old LC or CMNKO mice that were either fed or fasted for 24h. n=6:4:5:8. **(B)** Oil red O staining of heart sections from LC or CMNKO mice that were either fed or fasted for 24h. n=6:4:5:8. Scale bar: 50μm. Two-way ANOVA followed by Bonferoni post-tests was used for statistical analysis.

Appendix Table S1. Cardiac function measured by echocardiography in 2-month and 10-month old mice.

	2 months		10 months	
	**LC (n=9)**	**CMNKO (n=7)**	**LC (n=14)**	**CMNKO (n=10)**
**Heart Rate**	481.32±17.39	499.76±11.68	434.29±6.02	462.62±24.28
**Diameter;s**	1.69±0.07	1.68±0.08	1.93±0.05	1.96±0.05
**Diameter;d**	3.24±0.09	3.15±0.09	3.67±0.08	3.48±0.08
**Volume;s**	8.61±0.90	8.35±0.95	11.90±0.84	12.25±0.79
**Volume;d**	42.49±2.79	39.99±2.87	57.59±2.85	50.80±2.69
**Stroke Volume**	33.88±1.98	31.63±2.04	45.69±2.35	38.55±2.29[a]
**Ejection Fraction**	80.04±1.00	79.42±1.23	79.35±1.02	75.69±1.33[b]
**Fractional Shortening**	47.73±0.95	46.89±1.17	47.4±1.06	43.77±1.27[c]
**Cardiac Output**	20.99±4.00	18.41±3.23	18.41±1.18	17.40±1.74
**IVS;d**	0.94±0.05	0.93±0.06	1.01±0.04	1.09±0.05
**IVS;s**	1.55±0.04	1.41±0.08	1.64±0.04	1.64±0.06
**LVID;d**	3.19±0.06	3.21±0.11	3.65±0.1	3.48±0.11
**LVID;s**	1.74±0.10	1.74±0.10	1.97±0.08	2.06±0.07
**LVPW;d**	0.78±0.03	1.13±0.16	0.74±0.06	0.74±0.12
**LVPW;s**	1.19±0.05	0.41±0.16	1.28±0.06	1.37±0.07

a indicates p=0.0469; b, p=0.037; c, p=0.0392 vs 10 month LC group. Student's t test was used for statistical analysis.

Appendix Table S2. Cardiac function measured by echocardiography in LC, CMNKO and αMHC-Cre mice.

	LC (n=9)	CMNKO (n=11)	αMHC-Cre (n=7)
**Heart Rate**	521.20±5.45	506.89±11.12	540.25±4.78
**Diameter;s**	2.08±0.09	1.96±0.05	2.09±0.04
**Diameter;d**	4.02±0.11	3.43±0.07[a]	4.12±0.07[i]
**Volume;s**	14.58±1.52	12.25±0.77	14.36±0.74
**Volume;d**	71.75±4.56	48.90±2.50[b]	75.29±3.14[j]
**Stroke Volume**	57.17±3.55	36.65±1.79[c]	60.92±2.49
**Ejection Fraction**	79.90±1.40	75.06±0.51[d]	80.95±0.43[k]
**Fractional Shortening**	48.28±1.40	42.92±0.44[e]	49.19±0.44[l]
**Cardiac Output**	29.82±1.93	18.64±1.08[f]	32.91±1.35[m]
**IVS;d**	0.86±0.07	1.12±0.05[g]	1.04±0.14
**IVS;s**	1.34±0.06	1.34±0.07	1.43±0.05
**LVID;d**	3.73±0.12	3.25±0.10[h]	3.65±0.1[n]
**LVID;s**	2.15±0.09	1.98±0.06	2.17±0.05
**LVPW;d**	1.21±0.07	1.35±0.09	1.40±0.12
**LVPW;s**	1.32±0.09	1.54±0.09	1.38±0.13

a indicates p=0.0002; b, p=0.0002; c, p<0.0001; d, p=0.0025; e, p=0.0009; f, p<0.0001; g, p=0.0065; h, p=0.0066 vs LC group. i indicated p=0.5125; j, p=0.5582; k, p=0.5305; m, p=0.2367; n, p=0.6402 vs LC. Student's t test was used for statistical analysis.

Appendix Table S3. Cardiac function measured by echocardiography in LC and CMNKO mice with or without AAC.

	Sham		AAC	
	LC (n=9)	CMNKO (n=7)	LC (n=14)	CMNKO (n=12)
**Heart Rate**	469.80±16.98	503.87±11.36	457.67±7.79	473.82±15.13
**Diameter;s**	1.55±0.07	1.72±0.15	2.36±0.11[a]	3.34±0.20[b]
**Diameter;d**	3.07±0.07	3.18±0.17	3.65±0.10[c]	4.12±0.16[d]
**Volume;s**	6.86±0.81	9.39±2.44	20.45±2.64[e]	48.26±5.87[f]
**Volume;d**	37.31±2.13	41.41±5.95	57.20±3.79[g]	77.08±6.39[h]
**Stroke Volume**	30.45±1.50	32.02±3.35	36.75±1.65	28.83±2.03[i]
**Ejection Fraction**	81.96±1.39	78.84±1.96	65.52±2.10[j]	40.36±4.11[k]
**Fractional Shortening**	49.73±1.55	46.43±1.72	35.58±1.51[l]	19.93±2.45[m]
**Cardiac Output**	14.42±1.06	16.19±1.98	16.21±0.74	13.062±0.98
**IVS;d**	0.97±0.06	1.01±0.06	1.19±0.04	1.26±0.06
**IVS;s**	1.58±0.03	1.53±0.07	1.73±0.05	1.57±0.07
**LVID;d**	3.08±0.07	3.26±0.16	3.69±0.10	4.23±0.17
**LVID;s**	1.58±0.04	1.78±0.16	2.40±0.11[n]	3.41±0.21[o]
**LVPW;d**	0.75±0.03	1.00±0.20	1.06±0.06	1.09±0.07
**LVPW;s**	1.25±0.07	1.57±0.16	1.56±0.06	1.34±0.10

2-3 month old mice were subjected to Sham operation or AAC. Heart function was measured 2 weeks after surgery. a indicates p=0.0007; b, p<0.0001; c, p=0.0041; d, p=0.012; e, p=0.0331; f, p<0.0001; g, p=0.0125; h, p=0.0065; I, p=0.0098; j, p=0.0005; k, p<0.0001; l, p<0.0001; m, p<0.0001; n, p=0.001; o, p<0.0001. a, c, e, g, j, l and n vs Sham LC Group. b, d, f, h, I, k, m and o vs AAC LC group. Two-way ANOVA followed by Bonferoni post-tests was used for statistical analysis.

Appendix Table S4. Cardiac function measured by echocardiography in mice infected with AAV9 GFP or AAV9 NCoR1-RIDs.

	NO surgery		AAC 2 weeks		AAC 4 weeks	
	AAV9 GFP (n=6)	AAV9 NCoR1-RIDs (n=6)	AAV9 GFP (n=6)	AAV9 NCoR1-RIDs (n=6)	AAV9 GFP (n=6)	AAV9 NCoR1-RIDs (n=6)
**Heart Rate**	464.89±6.45	464.60±6.88	473.55±7.62	475.14±8.13	478.21±7.11	476.09±6.34
**Diameter;s**	2.07±0.33	2.16±0.09	2.67±0.15	2.71±0.18	3.04±0.09	2.37±0.16[a]
**Diameter;d**	3.34±0.19	3.38±0.16	3.72±0.17	4.10±0.16	3.75±0.08	3.19±0.16
**Volume;s**	14.44±2.23	15.79±1.65	27.08±3.82	28.42±5.31	36.53±2.54	20.24±3.23
**Volume;d**	46.67±6.00	47.55±5.35	60.00±6.84	74.96±7.49	60.36±3.15	41.39±4.82
**Stroke Volume**	32.23±3.98	31.77±3.83	32.93±3.63	46.54±2.73	23.83±1.50	21.15±1.75
**Ejection Fraction**	69.39±1.42	66.50±1.14	55.37±2.72	63.23±2.81[b]	39.62±1.91	52.31±2.38[c]
**Fractional Shortening**	38.03±1.08	35.83±0.95	28.32±1.73	34.04±1.90[d]	18.89±1.03	26.01±1.38[e]
**Cardiac Output**	14.97±1.81	14.78±1.85	15.66±1.90	22.15±1.47	11.37±0.66	10.03±0.74
**IVS;d**	1.06±0.12	0.97±0.11	1.16±0.07	1.18±0.07	1.04±0.08	1.20±0.10
**IVS;s**	1.34±0.06	1.36±0.08	1.44±0.15	1.30±0.07	0.90±0.04	0.99±0.06
**LVID;d**	3.20±0.20	3.46±0.15	3.70±0.21	3.77±0.12	3.81±0.08	3.22±0.13
**LVID;s**	2.15±0.19	2.46±0.11	2.71±0.19	2.67±0.16	3.13±0.10	2.36±0.13[f]
**LVPW;d**	0.92±0.12	1.00±0.13	1.47±0.07	1.04±0.07	1.06±0.08	1.10±0.09
**LVPW;s**	1.24±0.07	1.19±0.13	1.74±0.07	1.36±0.08	1.21±0.07	1.28±0.08

2 month old mice were subjected to Sham operation or AAC. Heart function was measured before, 2 weeks and 4 weeks after surgery. a indicates p=0.0049; b, p=0.0453; c, p=0.0007; d, p=0.0206; e, p=0.0032; f, p=0.0036. b and d vs AAV9 GFP AAC 2 weeks. a, c, e and f vs AAV9 GFP AAC 4 weeks. Two-way ANOVA followed by Bonferoni post-tests was used for statistical analysis.

Appendix Table S5. List of primer sequences for qRT-PCR

Name	Sequence	Species
GAPDH F	ATGTTCCAGTATGACTCCACTCACG	mouse
GAPDH R	GAAGACACCAGTAGACTCCACGACA	mouse
18s F	TTGATTAAGTCCCTGCCCTTTGT	mouse
18s R	CGATCCGAGGGCCTCACTA	mouse
Acta1 F	CCCAAAGCTAACCGGGAGAAG	mouse
Acta1 R	CCAGAATCCAACACGATGCC	mouse
Nppa F	GCTTCCAGGCCATATTGGAG	mouse
Nppa R	GGGGGCATGACCTCATCTT	mouse
Nppb F	ATGGATCTCCTGAAGGTGCTG	mouse
Nppb R	GTGCTGCCTTGAGACCGAA	mouse
Col1a1 F	GCTCCTCTTAGGGGCCACT	mouse
Col1a1 R	CCACGTCTCACCATTGGGG	mouse
Col1a2 F	GTAACTTCGTGCCTAGCAACA	mouse
Col1a2 R	CCTTTGTCAGAATACTGAGCAGC	mouse
Fn1 F	ATGTGGACCCCTCCTGATAGT	mouse
Fn1 R	GCCCAGTGATTTCAGCAAAGG	mouse
CTGF F	GGGCCTCTTCTGCGATTTC	mouse
CTGF R	ATCCAGGCAAGTGCATTGGTA	mouse
Tgfb3 F	CAGGCCAGGGTAGTCAGAG	mouse
Tgfb3 R	ATTTCCAGCCTAGATCCTGCC	mouse
NCoR1 F	CTGGTCTTTCAGCCACCATT	mouse
NCoR1 R	CCTTCATTGGATCCTCCATC	mouse
cJun-F	GCATTTGGAGAGTCCCTTCTC	mouse
cJun-R	TTAAGGCGCAGAAGAGATTTG	mouse
MCP-1 F	TTAAAAACCTGGATCGGAACCAA	mouse
MCP-1 R	GCATTAGCTTCAGATTTACGGGT	mouse
Glut4 F	GTGACTGGAACACTGGTCCTA	mouse
Glut4 R	CCAGCCACGTTGCATTGTAG	mouse
ACTA1 F	AGCAGCAGAAACTAGACACCA	Rat
ACTA1 R	CCACGATGGATGGGAACACA	Rat
Nppa F	CTTCTTCCTCTTCCTGGCCT	Rat
Nppb R	TTCATCGGTCTGCTCGCTCA	Rat
GAPDH F	GCGAGATCCCGCTAACATCA	Rat
GAPDH R	CTCGTGGTTCACACCCATCA	Rat
MEF2a F	AGAGGAACCGACAGGTGACT	Rat
MEF2a R	AGTGCTGGCGTACTGAAACA	Rat
MEF2c F	ACAAGCCAAATCTCCTCCCC	Rat
MEF2c R	ACATCCTCAGACACTGATGGC	Rat
MEF2d F	GCATCATTTGAACAATGCCCA	Rat
MEF2d R	GGCCCTGGCTGAGTAAACTT	Rat
18s F	CTTAGAGGGACAAGTGGCG	Human
18s R	ACGCTGAGCCAGTCAGTGTA	Human
Nppa F	GCAGGATGGACAGGATTGGAG	Human
Nppa R	GTCCTCCCTGGCTGTTATCT	Human
Myh7 F	GTCTTTCCCTGCTGCTCTCAG	Human
Myh7 R	GGGCTACTCAAGTGTGTCTAC	Human
Nppb F	CTTTCCTGGGAGGTCGTTCC	Human
Nppb R	GTTGCGCTGCTCCTGTAAC	Human
