# Supplementary material for: Nuclear receptor corepressor 1 represses cardiac hypertrophy
Source: EMBO Mol Med. 2019 Sep 18;11(11):e9127. doi: 10.15252/emmm.201809127 (PMC6835202; doi:10.15252/emmm.201809127)
Supplement: Supplementary file 2 — Expanded View Figures PDF [file EMMM-11-e9127-s002.pdf]

Expanded View Figures

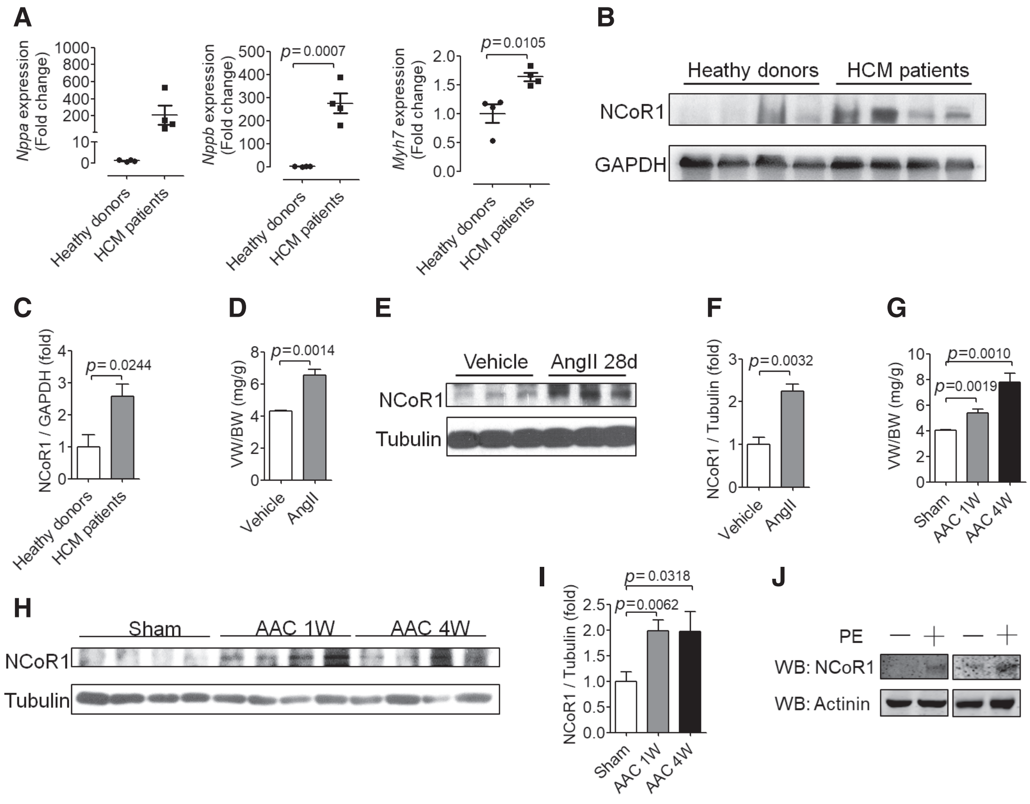

Figure EV1. The expression of nuclear receptor corepressor 1 (NCoR1) is elevated in hypertrophied hearts or cardiomyocytes.

- A Quantitative reverse transcriptase polymerase chain reaction (qRT–PCR) analysis of hypertrophy-related genes in heart samples from healthy donors or hypertrophic cardiomyopathy (HCM) patients. n = 4:4. *Nppa, natriuretic peptide type A*; *Nppb, natriuretic peptide type B*; *Myh7, myosin, heavy polypeptide 7, cardiac muscle, beta*.
- B Western blotting analysis of NCoR1 in heart samples from healthy donors or HCM patients.
- C Quantification of (B). n = 4:4.
- D Ventricular weight-to-body weight ratio (VW/BW) of C57BL/6 mice infused with vehicle (0.9% NaCl) or angiotensin II (AngII) for 4 weeks. n = 3:3.
- E Western blotting analysis of NCoR1 in ventricular samples from mice shown in (D).
- F Quantification of (E). n = 3:3.
- G VW/BW of C57BL/6 mice subjected to sham operation or abdominal aortic constriction (AAC) for 1 or 4 weeks. n = 4:4:4.
- H Western blotting analysis of NCoR1 in ventricular samples from mice shown in (G).
- I Quantification of (H). n = 4:4:4.
- J Western blotting analysis of NCoR1 in neonatal rat ventricular myocytes treated without or with phenylephrine (PE) for 24 h.

Data information: Data are presented as mean ± SEM. Student's t-test was used for statistical analysis.
Source data are available online for this figure.

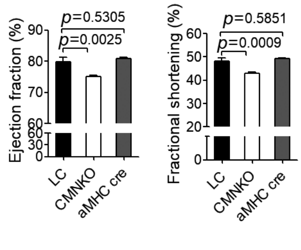

Figure EV2. αMHC-Cre did not affect cardiac function in 10-month-old mice.

Ejection fraction and fractional shortening measured by echocardiography. LC, littermate control mice; CMNKO, cardiomyocyte-specific NCoR1 knockout mice. n = 9:11:7. Data are presented as mean ± SEM. Student's t-test was used for statistical analysis.

© 2019 The Authors

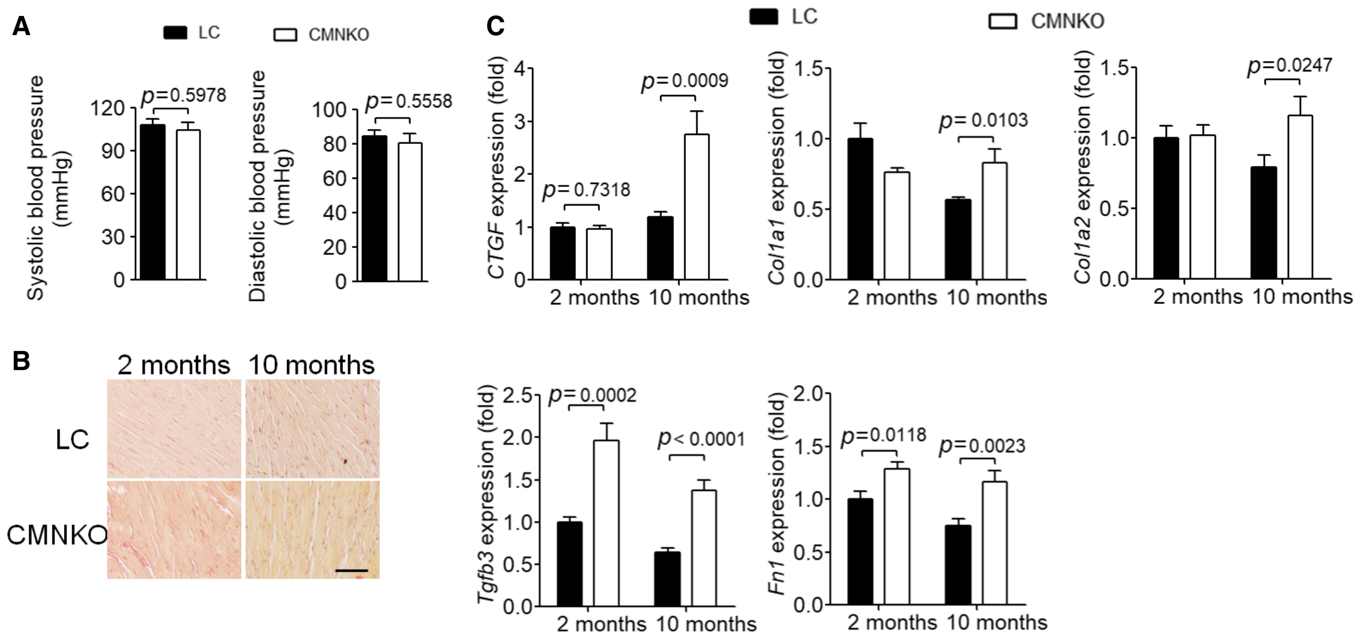

Figure EV3. NCoR1 deficiency in cardiomyocytes does not affect blood pressure or cardiac fibrosis.

A Systolic blood pressure and diastolic blood pressure were monitored using non-invasive tail-cuff system in 2-month-old mice. n = 6:6.

B Picrosirius red staining of cross sections of left ventricles of LC or CMNKO mice. Scale bar: 50 µm.

C qRT–PCR analysis of fibrosis-related genes in left ventricles of LC or CMNKO mice. n = 9:9:11:8. CTGF indicates connective tissue growth factor precursor; Fn1, fibronectin 1; TGFβ3, transforming growth factor β 3; Col1a1, collagen, type I, alpha 1; Col1a2, collagen, type I, alpha 2.

Data information: Data are presented as mean ± SEM. Student's *t*-test was used for statistical analysis.

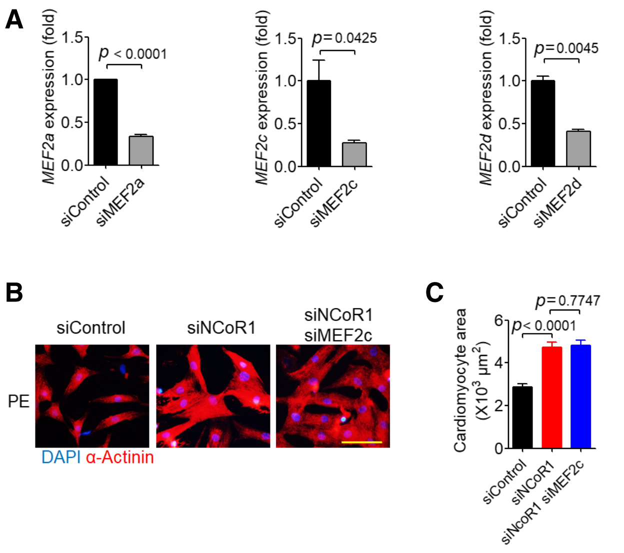

Figure EV4. MEF2c does not mediate the effects of NCoR1 on PE-induced cardiomyocyte hypertrophy.

A Knockdown efficiency of MEF2a, MEF2c, and MEF2d in NRVMs. siControl indicates control siRNA; siMEF2a indicates MEF2a siRNA; siMEF2c, MEF2c siRNA; siMEF2d, MEF2d siRNA. n = 3.

B Representative immunofluorescence staining of α-Actinin in NRVMs transfected with siRNA for 48 h and then treated with PE for another 48 h. Scale bar: 50 µm.

C Quantification of the surface area of α-Actinin-positive NRVMs with or without knockdown of NCoR1 and/or MEF2c. A total of 30 NRVMs were randomly chosen from three replicate coverslips for each group and used for statistical analysis.

Data information: Data represent three independent experiments. Data are presented as mean ± SEM. Student's *t*-test was used for statistical analysis.

© 2019 The Authors

Figure EV5. NCoR1 affects subcellular translocation of HDACs but does not directly interact with them in NRVMs.

A Representative immunofluorescence staining of HDAC4-GFP in NRVMs. Cells were infected by HDAC4-GFP adenovirus and then control lentivirus (Control) or NCoR1-flag lentivirus (NCoR1-OV), and subsequently treated with vehicle (H_2O) or PE. Scale bar: 20 μm.
B Quantification of cytosolic accumulation of HDAC4-GFP. $n = 5$.
C Representative immunofluorescence staining of HDAC5-GFP in NRVMs. Cells were infected by HDAC5-GFP adenovirus and then control lentivirus (Control) or NCoR1-flag lentivirus (NCoR1-OV), and subsequently treated with vehicle or PE. Scale bar: 20 μm.
D Quantification of cytosolic accumulation of HDAC5-GFP. $n = 5$.
E Co-IP analysis detecting interactions between NCoR1 and HDAC4, HDAC5 or HDAC9 in NRVMs. Cells were infected with NCoR1-flag lentivirus and then HDAC4 adenovirus, HDAC5-GFP adenovirus, or HDAC9 adenovirus.
F Co-IP analysis showing interactions between NCoR1, MEF2a, and HDAC4 in ventricular samples.
G Co-IP analysis detecting interactions between NCoR1, MEF2a, and HDAC5 in NRVMs. Cells were infected with NCoR1-flag lentivirus and then MEF2a-HA lentivirus and HDAC5-GFP adenovirus.

Data information: Data represent three independent experiments. Data are presented as mean ± SEM. Two-way ANOVA followed by Bonferroni post-tests was used for statistical analysis.
Source data are available online for this figure.

© 2019 The Authors

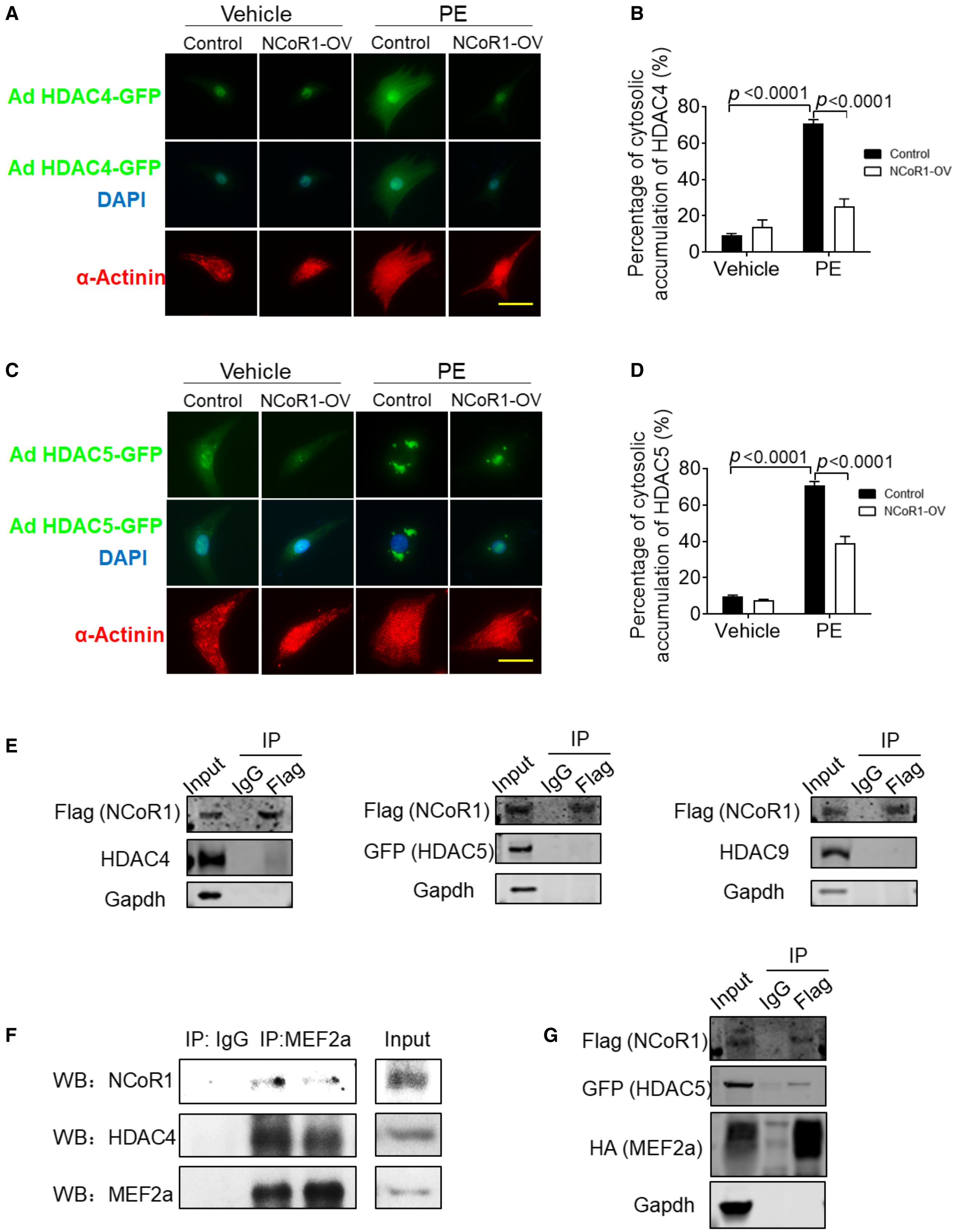

Figure EV5.

© 2019 The Authors
