# Supplementary figures and images for: Nuclear receptor corepressor 1 represses cardiac hypertrophy
Source: EMBO Mol Med. 2019 Sep 18;11(11):e9127. doi: 10.15252/emmm.201809127 (PMC6835202; doi:10.15252/emmm.201809127)

Figure 3A

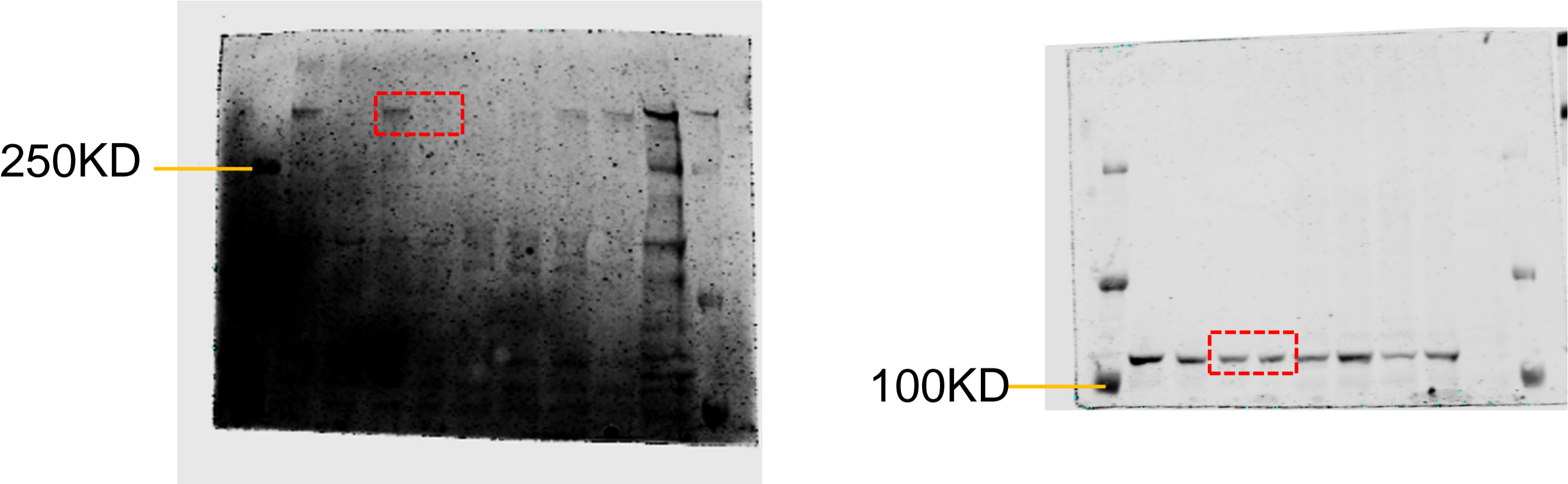

Figure 3E

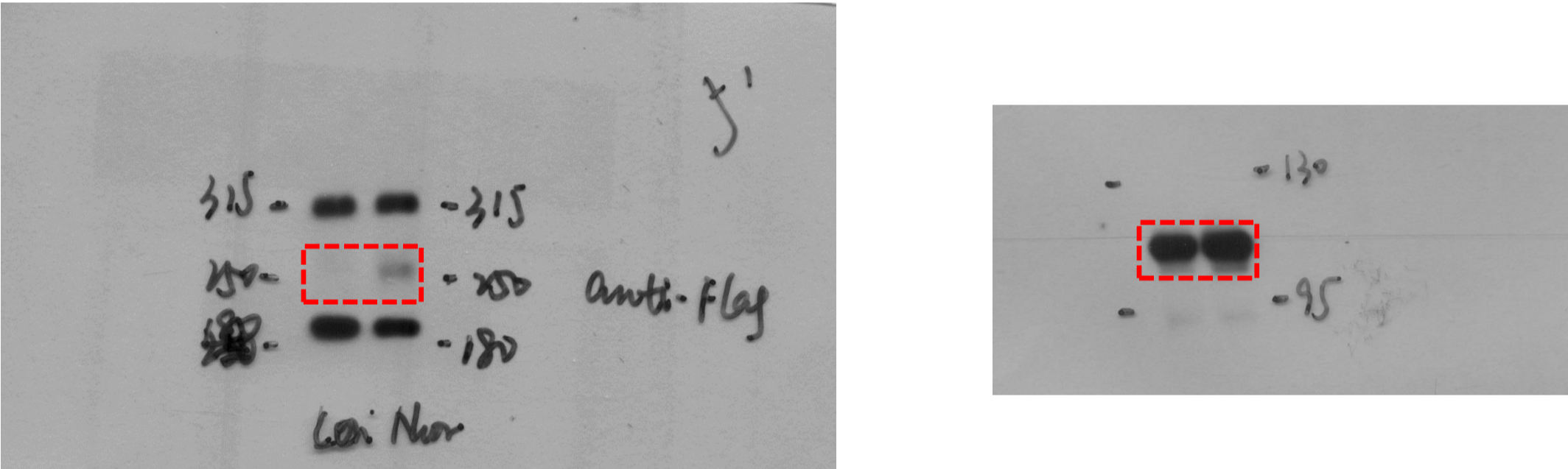

Supplement: Supplementary file 6 — Source Data for Figure 3 [file EMMM-11-e9127-s004.pdf]

Figure 5B

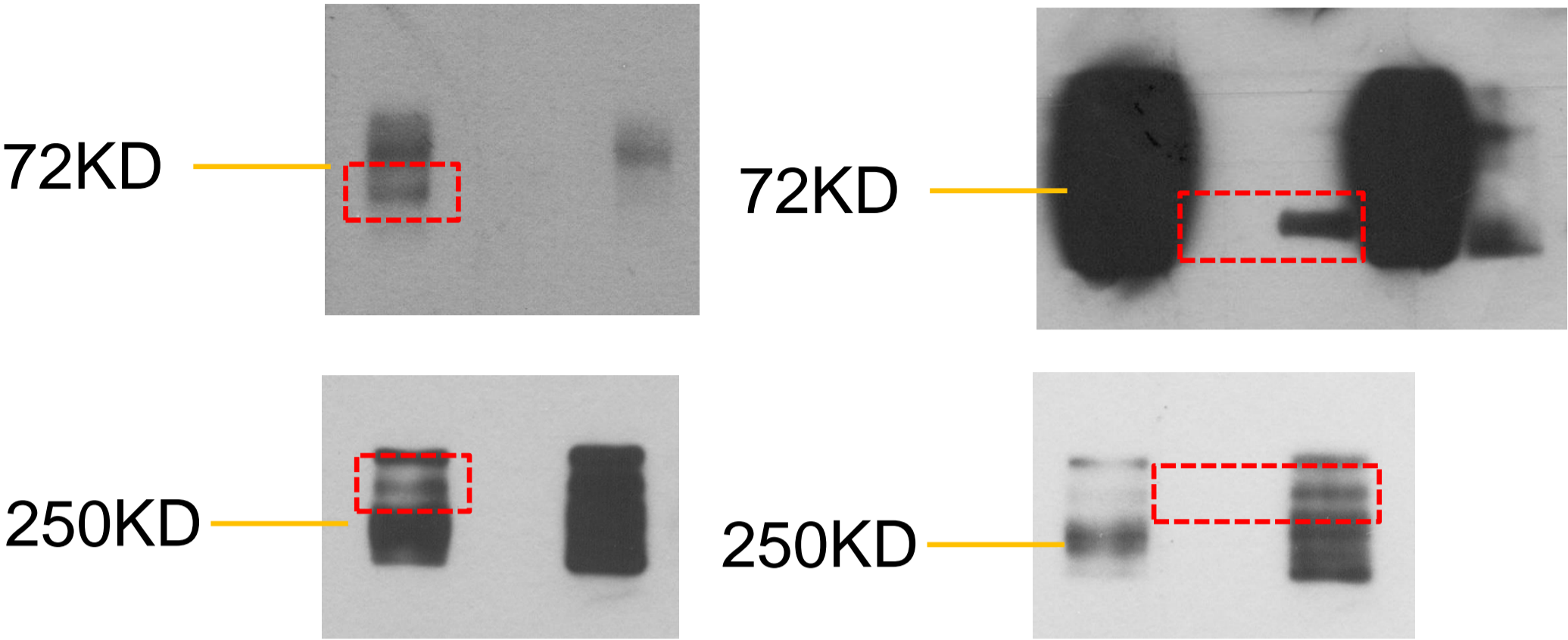

Figure 5C

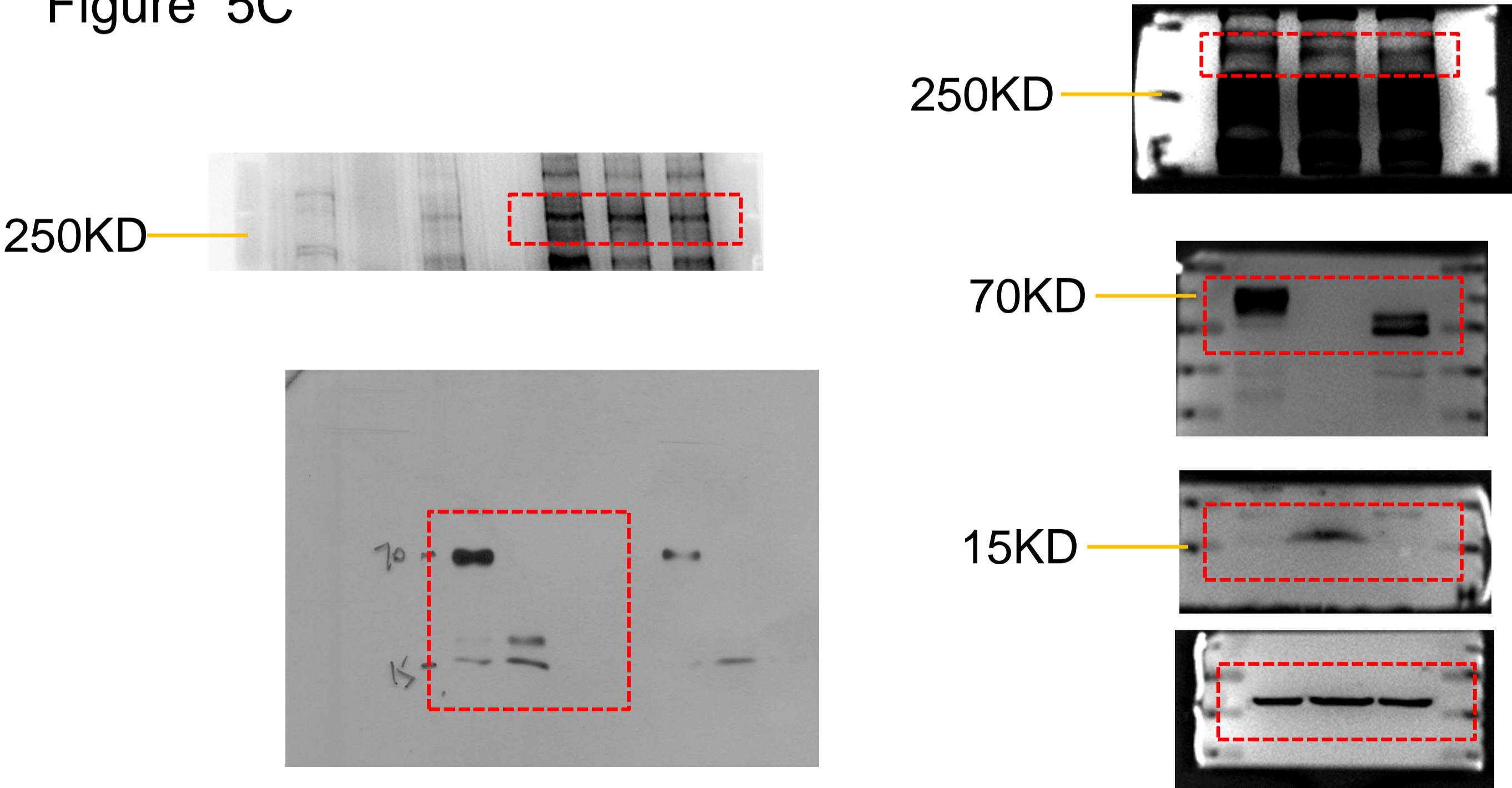

Figure 5D

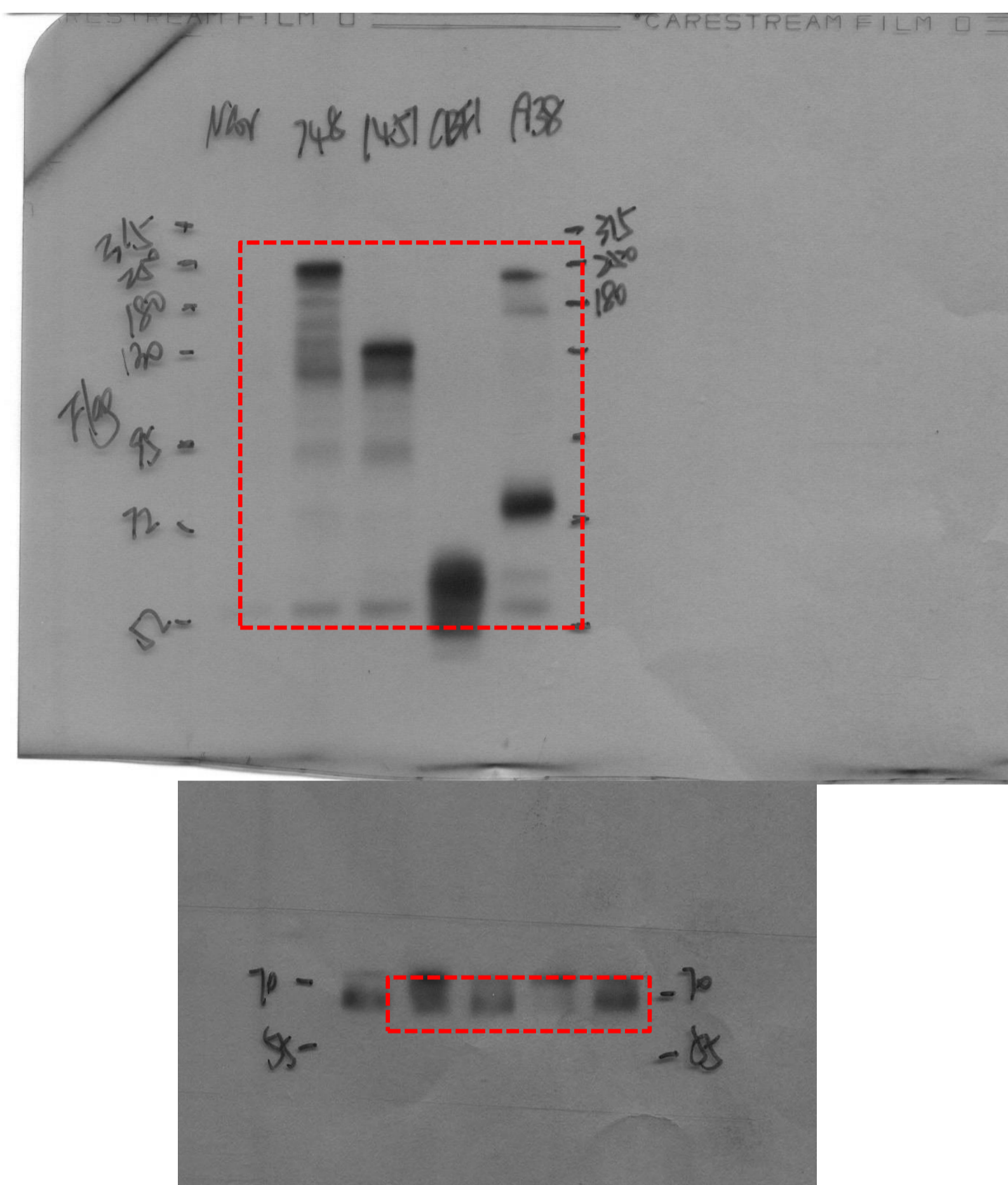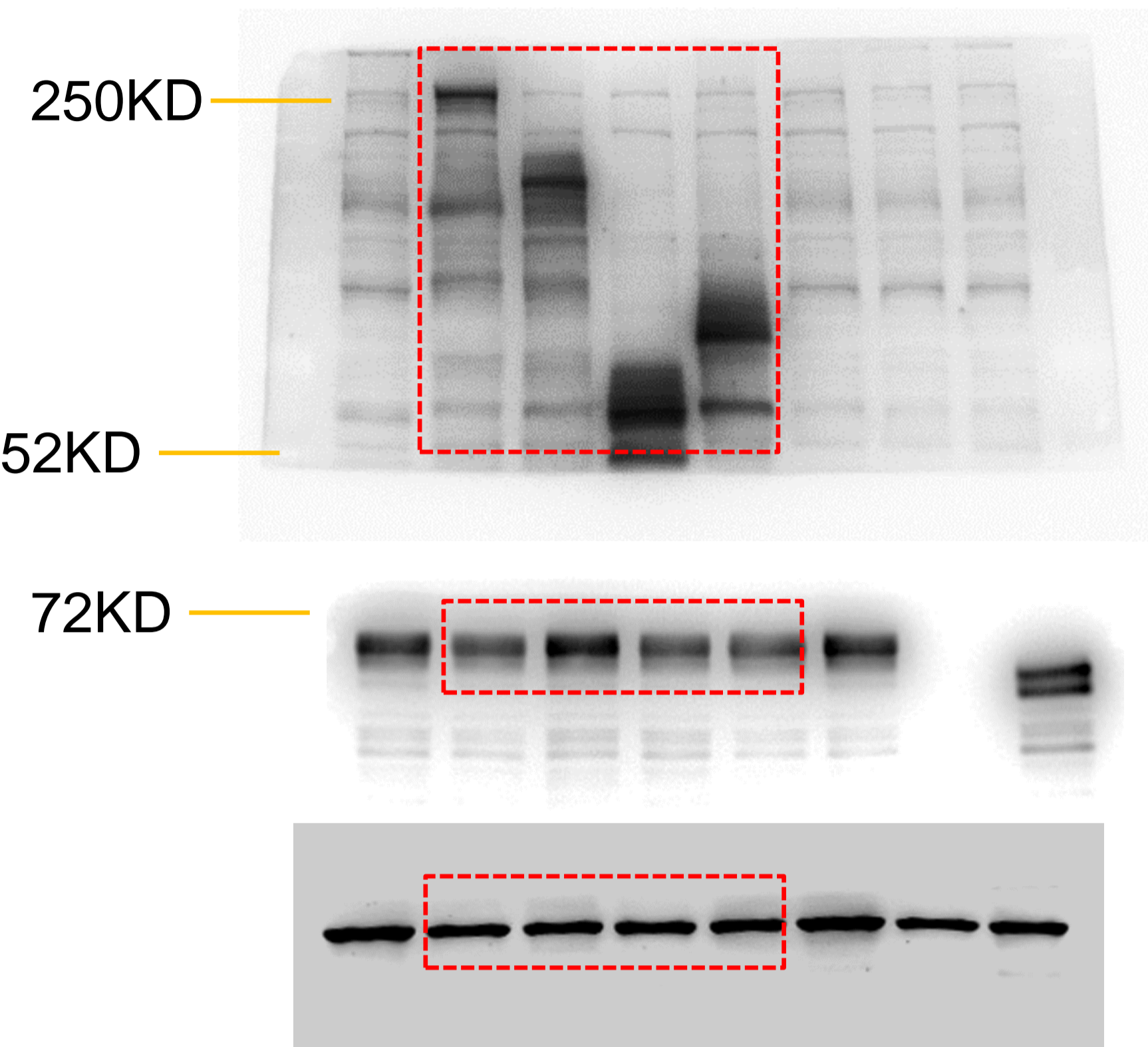

Figure 5F

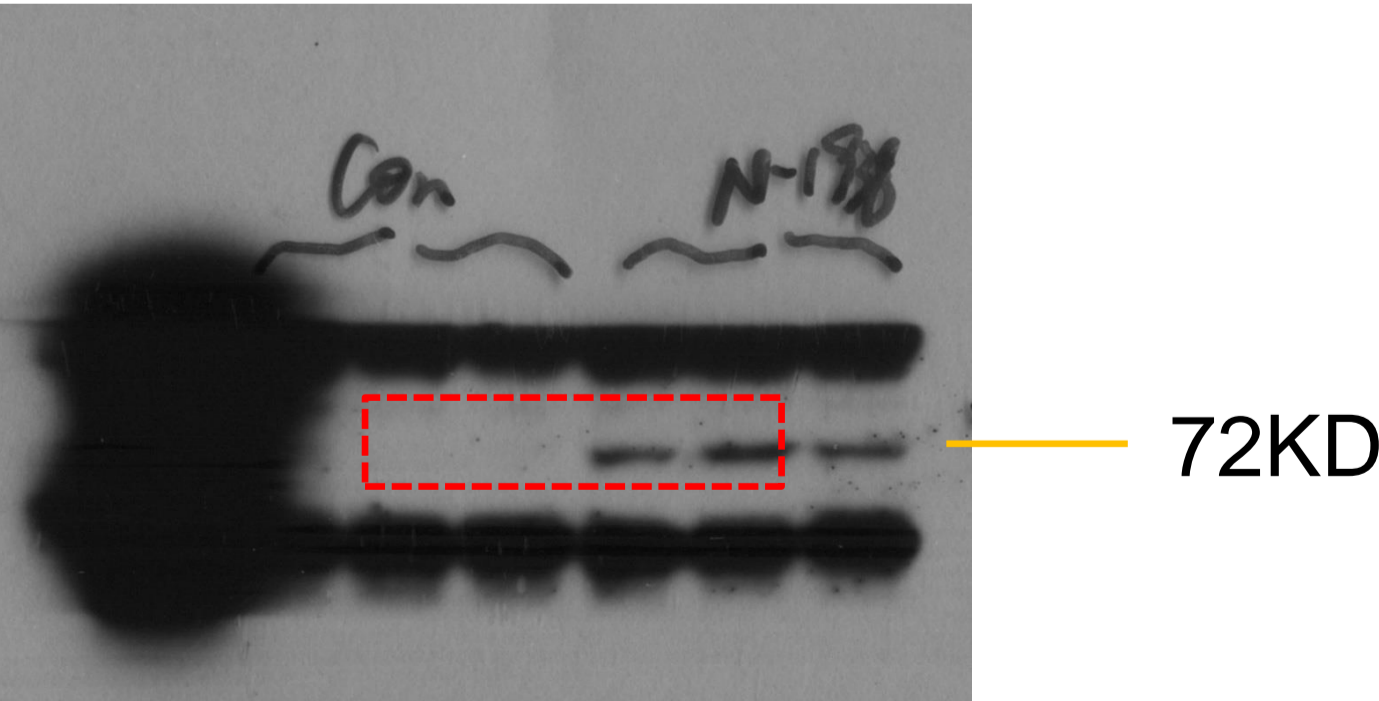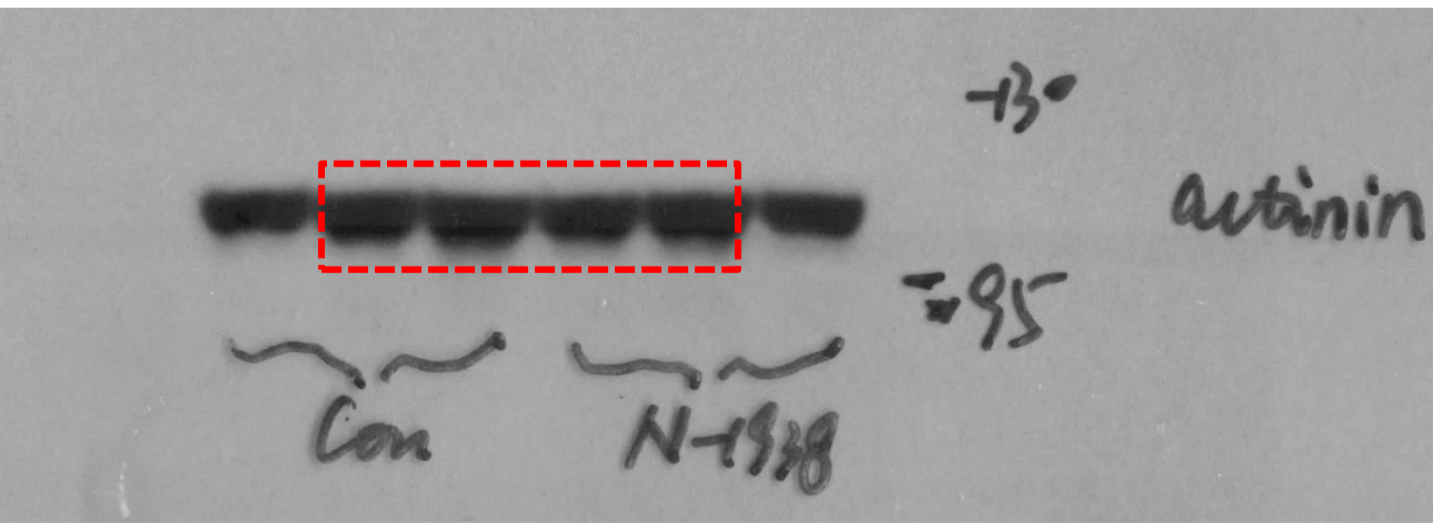

Supplement: Supplementary file 7 — Source Data for Figure 5 [file EMMM-11-e9127-s005.pdf]

Figure 7A

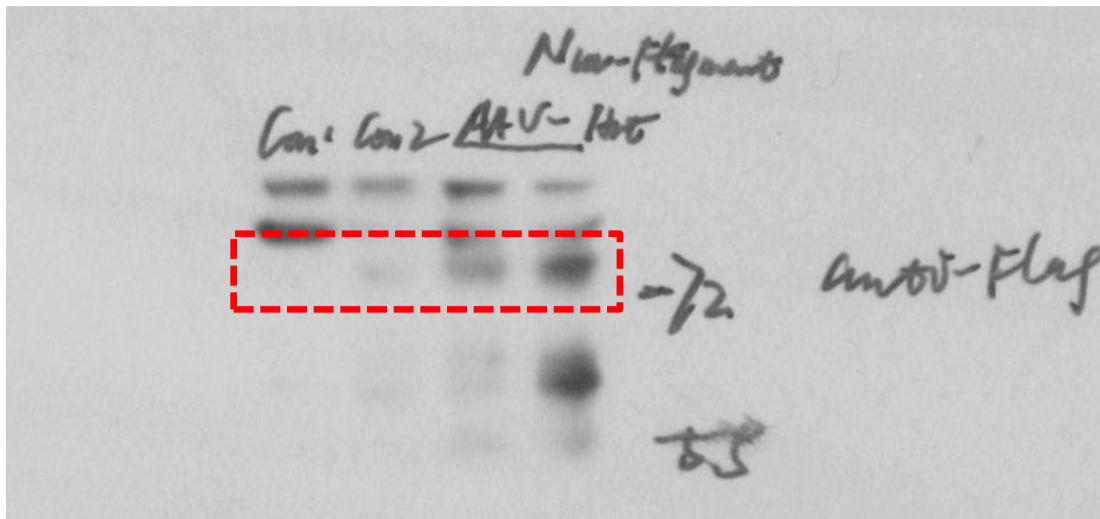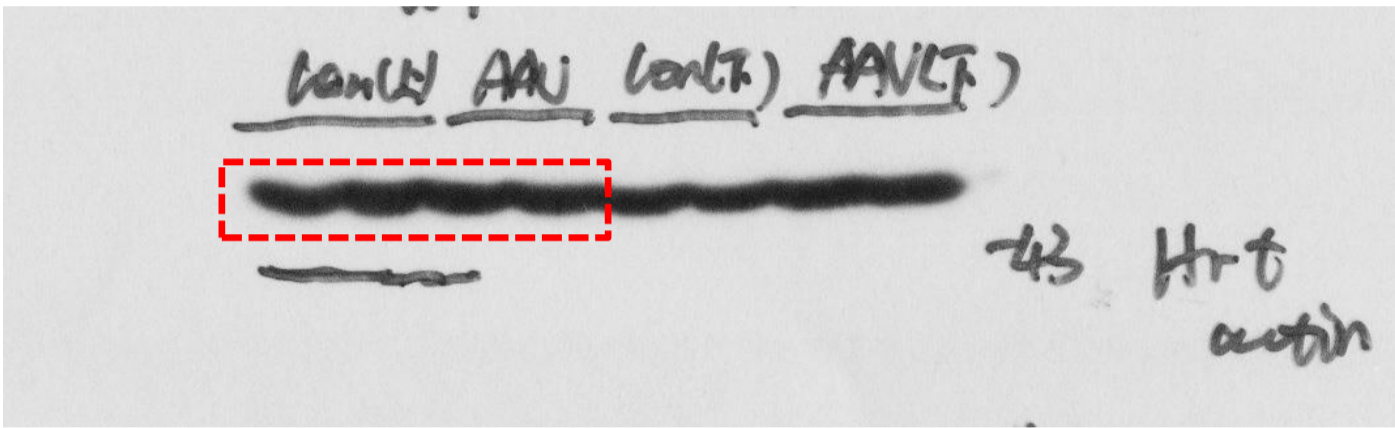

Supplement: Supplementary file 8 — Source Data for Figure 7 [file EMMM-11-e9127-s006.pdf]
