# Supplementary material for: Nuclear receptor corepressor 1 represses cardiac hypertrophy
Source: EMBO Mol Med. 2019 Sep 18;11(11):e9127. doi: 10.15252/emmm.201809127 (PMC6835202; doi:10.15252/emmm.201809127)

Figure EV1B

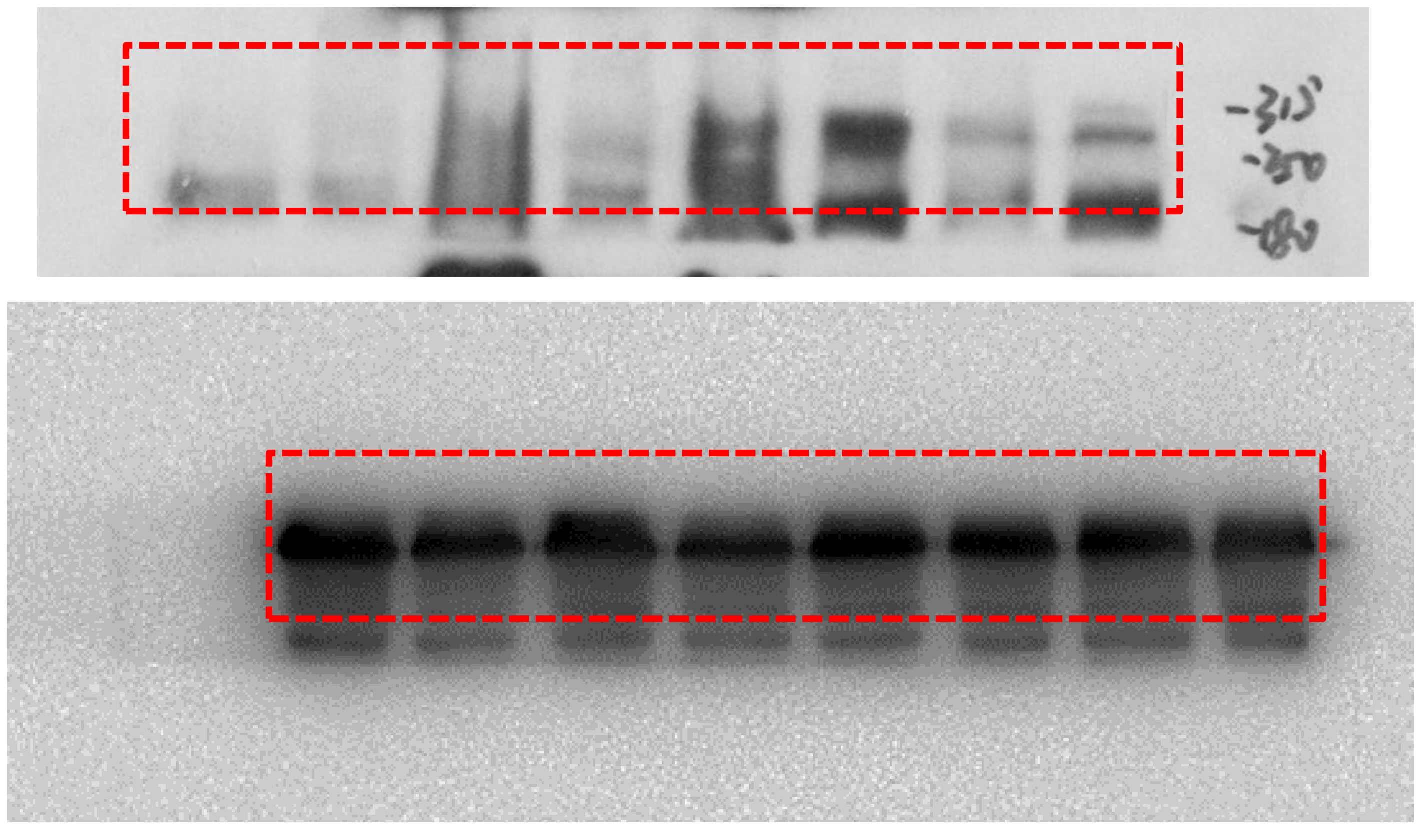

Figure EV1E

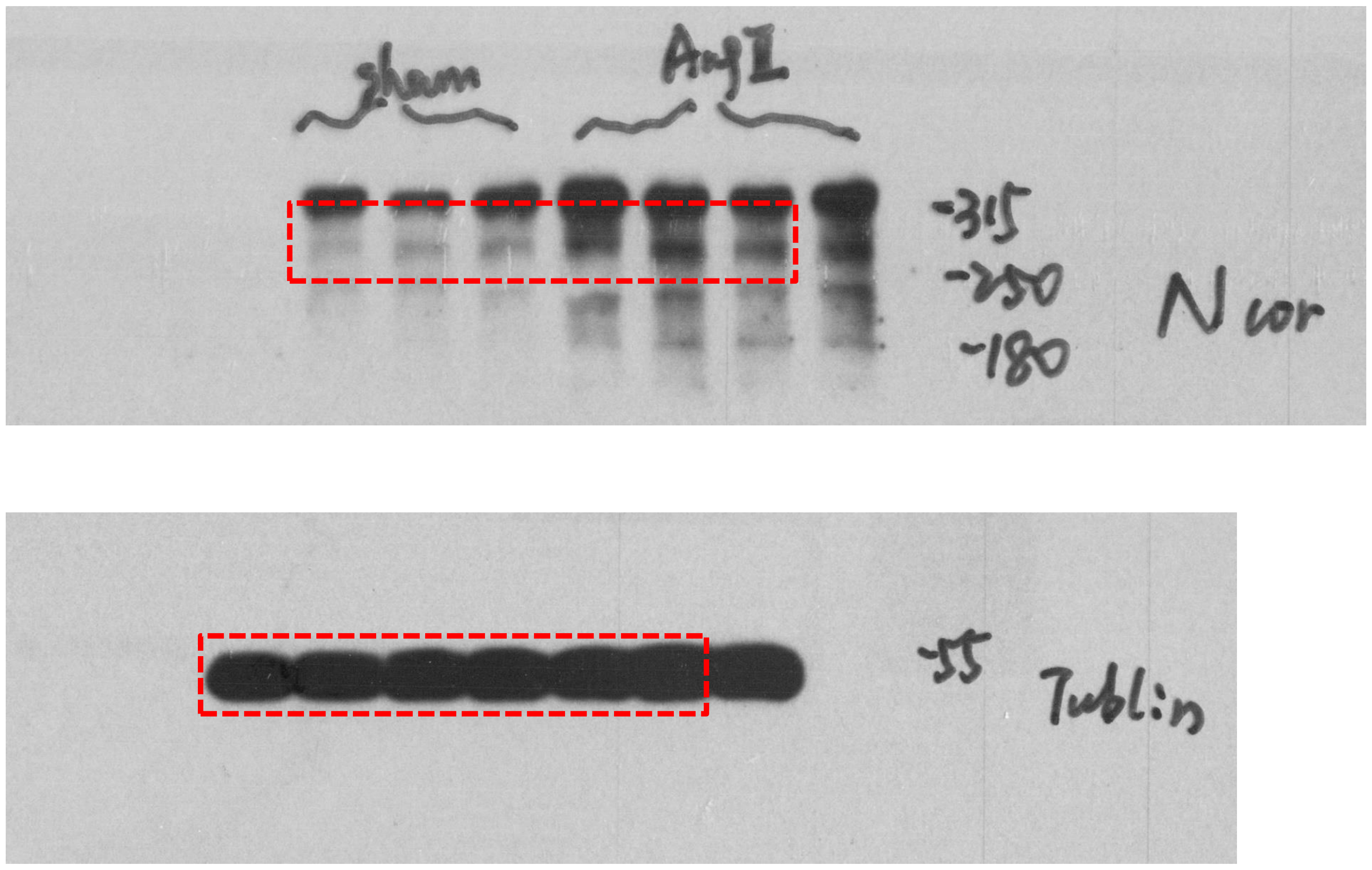

Figure EV1H

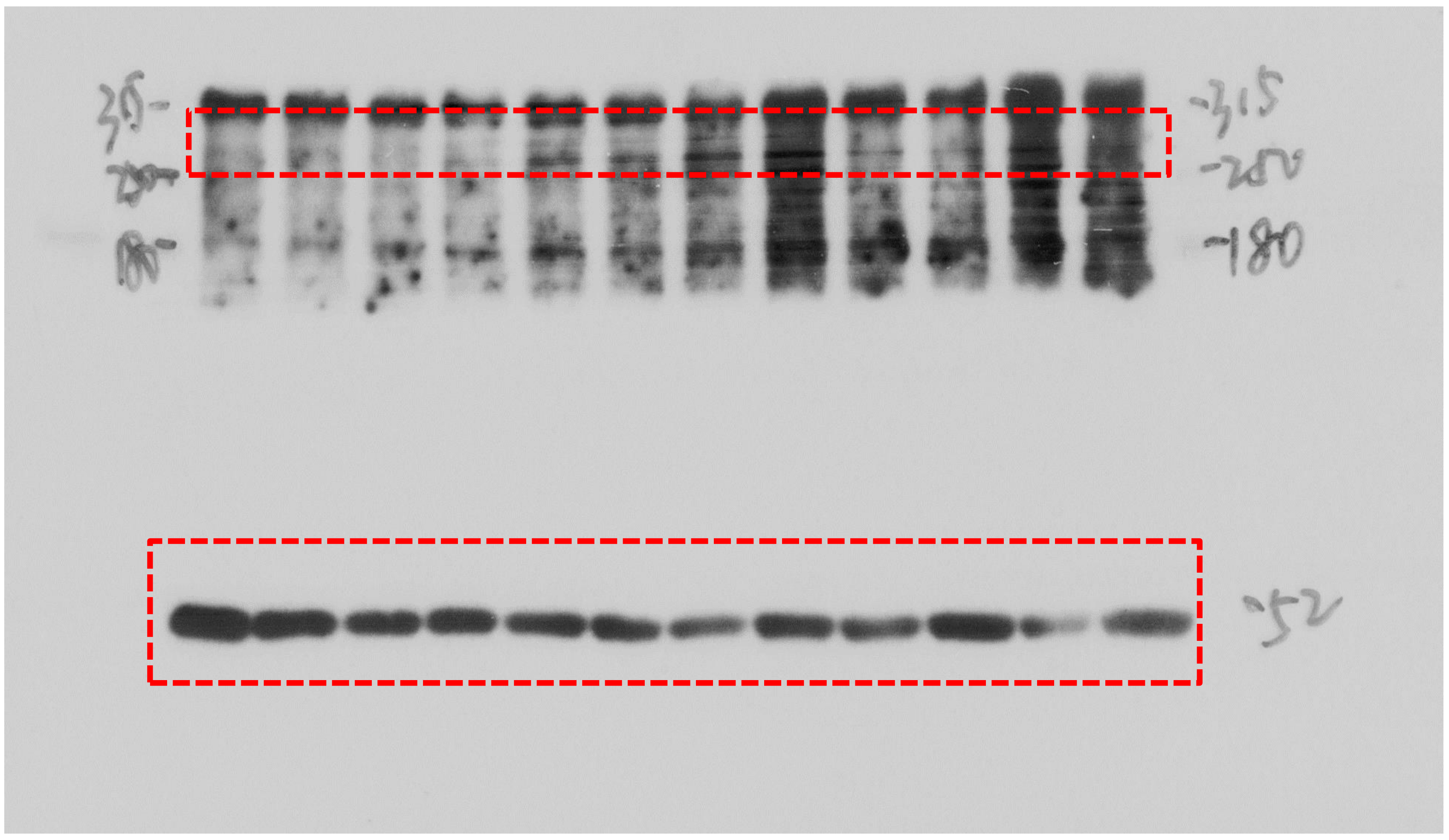

Figure EV1G

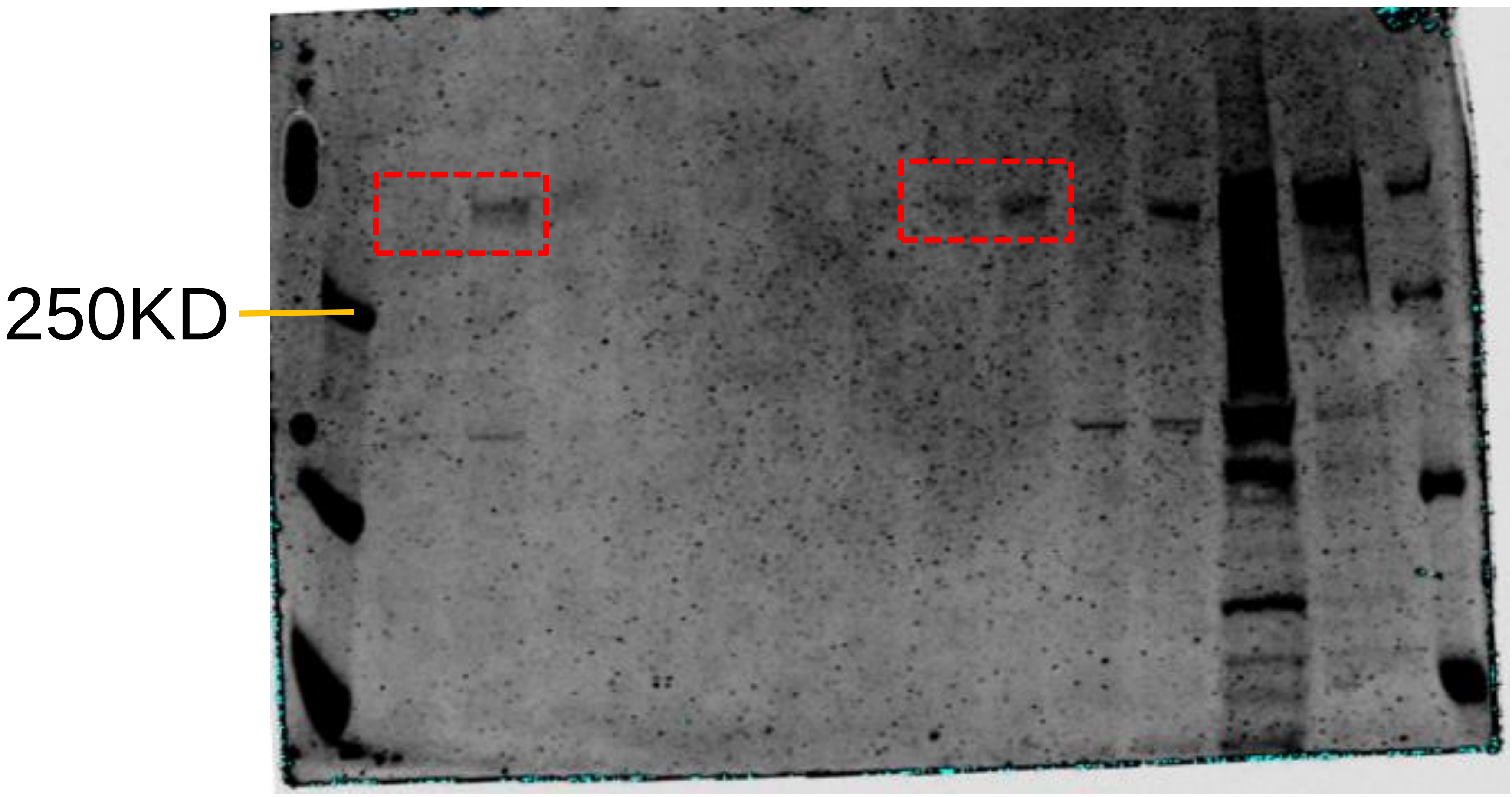

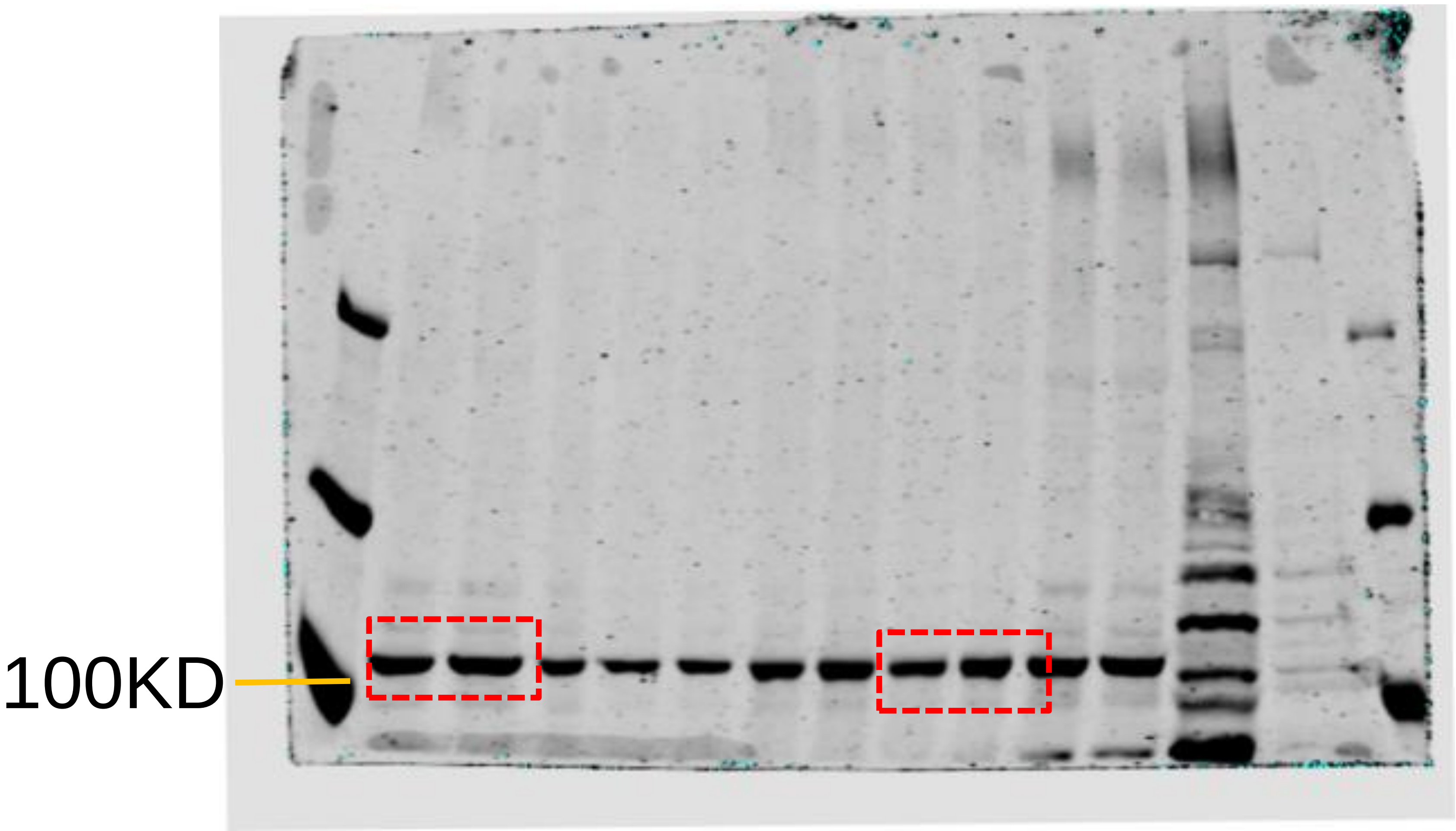

Supplement: Supplementary file 4 — Source Data for Expanded View and Appendix [file EMMM-11-e9127-s007.zip › emmm201809127-sup-0008-SDataEVFigures.zip!/Source_Data_For_Figure_EV1.pdf]
